# The efficacy and safety of fingolimod plus standardized treatment versus standardized treatment alone for acute ischemic stroke: A systematic review and meta‐analysis

**DOI:** 10.1002/prp2.972

**Published:** 2022-05-18

**Authors:** Peng Bai, Runxiu Zhu, Ping Wang, Feng Jiang, Jin Zhen, Yuan Yao, Chenhui Zhao, Zihong Liang, Meiling Wang, Bin Liu, Min Li, Na Li, Jun Yuan

**Affiliations:** ^1^ Department of Neurology Inner Mongolia People's Hospital No. 20 of Zhaowuda Road Hohhot 010017 Inner Mongolia People’s Republic of China; ^2^ Department of Neurosurgery The First Affiliated Hospital of Wannan Medical College Wuhu China; ^3^ Department of Psychiatry Inner Mongolia People's Hospital No. 20 of Zhaowuda Road Hohhot 010017 Inner Mongolia People’s Republic of China; ^4^ Nursing Department Inner Mongolia People's Hospital No. 20 of Zhaowuda Road Hohhot 010017 Inner Mongolia People’s Republic of China

**Keywords:** acute ischemic stroke, fingolimod, meta‐analysis, modified rankin scale

## Abstract

Acute ischemic stroke (AIS) is the most common type of stroke. Fingolimod is a sphingosine analog that acts on sphingosine‐1‐phosphate receptors (S1PR). Recently, the safety and efficacy of fingolimod in both patients with intracerebral hemorrhage and patients with AIS have been investigated in proof‐of‐concept trials. In this review, we performed a meta‐analysis to evaluate the efficacy and safety of fingolimod for AIS. This study was conducted according to the PRISMA (Preferred Reporting Items for Systemic review and Meta‐Analysis) statement. We searched for publications on the PubMed, Embase, Cochrane Central Register of Controlled Trials, Clinical trials, CNKI, Wanfang Data, VIP, CBM up to August 2021. We compiled five studies; a main meta‐analysis forest plots were conducted for the values of the proportion of patients whose modified Rankin scale (MRS) score was 0–1 at day 90. There were heterogeneities in each study; the method of sensitivity analysis was performed. A sensitivity analysis was performed with a mean difference (MD) of the efficacy of fingolimod plus standardized treatment versus standardized treatment alone. Random effect model is used for meta‐analysis regardless of the I^2^ index. The analysis was carried out for categorical variables using the risk ratio (RR), LogRR, and its 95% CI. The methodological quality of each randomized controlled trial (RCTs) was assessed according to the Cochrane Collaboration tool to assess the risk of bias (ROB). A meta‐analysis of five studies with 228 participants was conducted. The risk ratio of patients whose MRS score was 0–1 at day 90 between fingolimod plus standardized treatment and standardized treatment alone was 2.59 (95%CI, 1.48–4.56). The Fingolimod plus standard treatment group decreased infarct growth and improved clinical function than the standard treatment.

## Graphical abstract

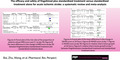

**Abbreviations**
AEs: adverse events
AIS: Acute ischemic stroke
BBB: blood‐brain barrier
BL: Bin Liu
CI: confidence interval
CNS: Central nervous system
CZ: Chenhui Zhao
FJ: Feng Jiang
JY: Jun Yuan
JZ: Jin Zhen
MD: mean difference
MeSH: Medical Subject Heading
ML: Min Li
MRS: Modified Rankin Scale
MS: multiple sclerosis
MW: Meiling Wang
NIHSS: National Institutes of Health Stroke Scale
NL: Na Li
PB: Peng Bai
PEDro: The Physiotherapy Evidence Database
PRISMA: Preferred Reporting Items for Systemic Reviews and Meta‐Analysis
PW: Ping Wang
RCTs: Randomized controlled trials
RR: risk ratio
RZ: Runxiu Zhu
S1PR: sphingosine‐1‐phosphate receptors
SAEs: serious adverse events
tPA: tissue plasminogen activator
YY: Yuan Yao
ZL: Zihong Liang

## 1 INTRODUCTION

Acute ischemic stroke (AIS) is the most common type of stroke. It has the characteristics of high morbidity, high mortality, and high disability, which seriously endangers the health and life of patients.^1^ Effective treatment after AIS will directly affect the prognosis of patients.^1^

Disabling stroke outcomes make it the second leading cause of death worldwide after cardiac ischemia. Therapy for AIS centers first on rapid revascularization of arterial territories, with additional focus on managing blood pressure and cerebral edema.^2^ Revascularization is currently achieved by the intravenous administration of tissue plasminogen activator (tPA) and intravascular therapy. However, the benefit of tPA is highly time dependent, considering that pooled analysis has documented loss of benefit beyond 4.5 h from onset of symptoms.^2^, ^3^, ^4^ Although numerous neuroprotective clinical trials have been conducted, no significant breakthrough has been made to improve the outcome of stroke patients.^2^, ^5^, ^6^

Cerebral ischemia‐induced cell death swiftly activates the immune system and initiates inflammation within the brain.^7^, ^8^, ^9^, ^10^, ^11^ In an early phase, these immune responses appear to exacerbate neurovascular dysfunction by promoting thrombus formation and accumulation of blood components in the cerebral microvasculature.^11^, ^12^, ^13^ These changes subsequently exacerbate the ischemic cascade catalyzing neural cell death in the penumbra, resulting in the extension of infarction, which potentially limits the efficacy of pharmacologic or mechanical reperfusion.^11^, ^14^, ^15^, ^16^

Fingolimod is a sphingosine analog that acts on sphingosine‐1‐phosphate receptors (S1PR). It was approved by the US. Food and Drug Administration in 2010 as the first oral disease‐modifying therapy for the relapsing–remitting form of multiple sclerosis (MS).^17^, ^18^ Fingolimod inhibits the egress of lymphocytes from lymph nodes and limits their recirculation.^18^, ^19^ Additional effects on the integrity of the blood–brain barrier (BBB) and direct action on neurons and glia that bear sphingosine‐1‐phosphate receptor may also contribute to its beneficial attributes in MS.^18^, ^20^, ^21^, ^22^ Recently, the safety and efficacy of fingolimod in both patients with intracerebral hemorrhage and patients with AIS have been investigated in proof‐of‐concept trials.^2^, ^18^ Fingolimod limited the expansion of infarct volume and ameliorated hemorrhagic transformation in patients with acute ischemic stroke who received intravenous alteplase within 4.5 h after stroke onset,^11^, ^18^ Meanwhile, in patients with acute anterior circulation occlusion who are >4.5 h after disease onset, fingolimod significantly improved the clinical outcome, reduced secondary lesion growth, and decreased microvascular permeability.^18^ In this systematic review, we performed a meta‐analysis to evaluate the efficacy and safety of fingolimod for acute ischemic stroke.

## 2 MATERIALS AND METHODS

### 2.1 Protocol and registration

Our protocol was registered prospectively with the Prospero website (CRD42021272343), the prospective international register of systematic reviews available at https://www.crd.york.ac.uk/prospero/display_record.php?ID=CRD42021272343.

### 2.2 Literature search

**FIGURE 1:**
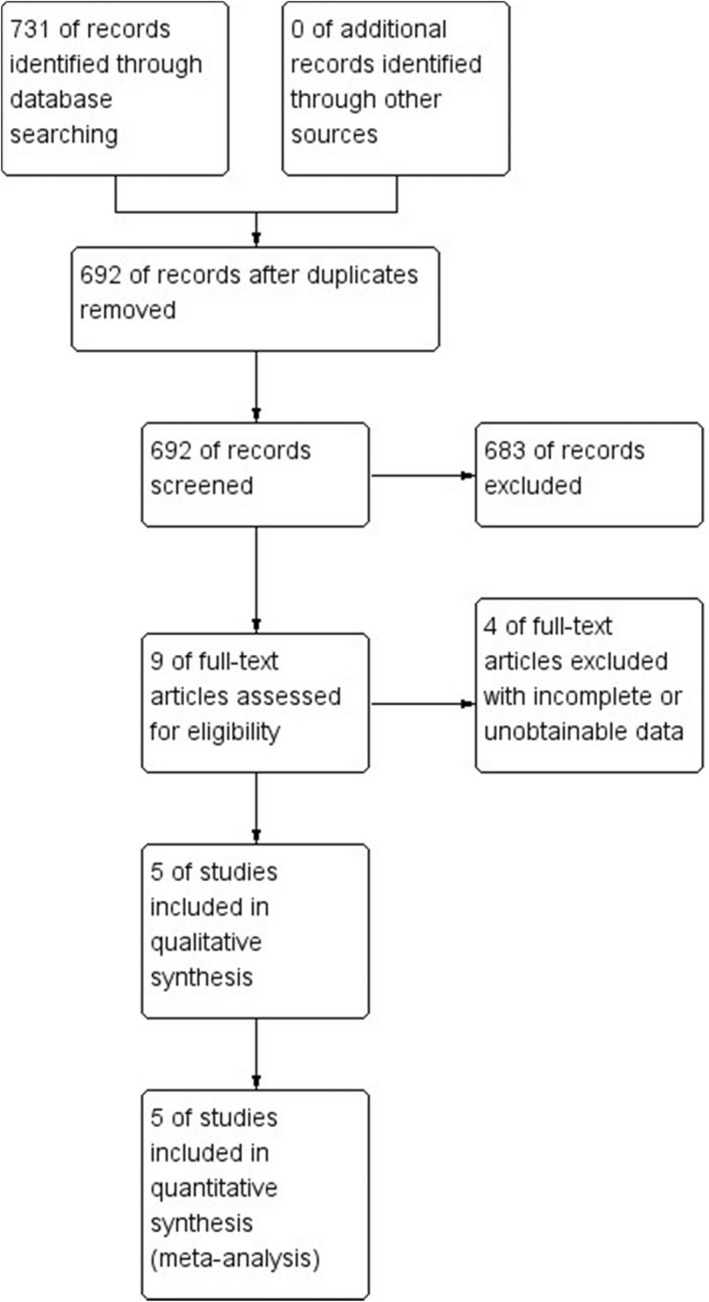
Flow chart presenting the process of the study selection for fingolimod meta‐analysis.

This search was restricted only to articles published in the English and Chinese language. We searched for publications on the PubMed, Embase, Cochrane Central Register of Controlled Trials, Clinical trials, CNKI, Wanfang Data, VIP, CBM up to August 2021. We did keyword and Medical Subject Heading (MeSH) searches for our theme, and MeSH terms, keywords, and their synonyms related to "Fingolimod hydrochloride" and "Cerebrovascular Disorders." A flowchart of the search strategy is shown in Figure 1. One of us used a standardized form of data extraction to extract data; another person checked it, revisited the data that did not match, and resolved the differences through discussion and consensus.

### 2.3 Inclusion and exclusion criteria for the literature

Studies were included if they fulfilled the following criteria: (1) Published English and Chinese randomized controlled trial in various journals regardless of whether the blind method was used or not; (2) >18 years of age; (3) acute onset of focal neurological deficit consistent with acute ischemic stroke; (4) fingolimod was given 0.5 mg of the drug orally once daily, for three consecutive days plus standardized treatment in the test group, standardized treatment was given in control group (standard treatment adhered to current American Heart Association guidelines including the intravenous administration of tPA, intravascular therapy, antiplatelet drugs, and so on). Exclusion criteria: (1) Case reports and studies that included fewer than two patients, review, meta‐analysis; (2) studies from which no data are provided or data are otherwise not extractable; (3) preexisting neurologic disability (a score greater than 2 on the MRS); (4) for studies published in more than one report, the most comprehensive and up‐to‐date version will be used.

### 2.4 Main variables

Among the five articles selected, we extracted the values of the proportion of patients whose MRS score was 0–1 at day 90, the mean difference in the change in MRS score at day 90, the change in the National Institutes of Health Stroke Scale (NIHSS) score at 24 h, the change in NIHSS score at day 7, the change in NIHSS score at day 90, relative infarct lesion growth at 24 h, relative infarct lesion growth at day 7, the incidence of complications/adverse events.

### 2.5 Data abstraction

The titles and abstracts of studies retrieved during the searches were screened for duplicates by two independent reviewers (PB and PW). Potentially relevant full texts were then screened according to our inclusion and exclusion criteria. The final included studies were then collated, and the two reviewers used standardized data extraction formats to extract the data. After extraction, both reviewers matched their data with each other and revisited papers where disagreements arose. Any discrepancies were resolved through discussion with other team members. The extracted data included the following: first author, study design, site of study, year of publication, language, number of patients receiving fingolimod, the values of variables. If required data were missing, not reported in the paper, or reported in an unusual form, the corresponding authors of the respective papers were contacted for clarification. Supplementary material associated with the main paper was also explored in such cases.

### 2.6 Risk of bias assessment and quality of evidence

Two authors (PB and PW) individually assessed the methodological quality of RCTs using the Cochrane Collaboration tool for assessing the risk of bias.^2324^ The criteria were selected a priori and included: (1) random sequence generation, (2) allocation concealment, (3) blinding of participants, (4) blinding of outcome assessment, (5) incomplete outcome data, (6) selective reporting (including reporting of all outcomes and specifying a primary outcome), and (7) other bias. The evaluated domains were judged as low risk, high risk, or unclear bias per established criteria. In the case of evaluation discrepancies, the authors discussed and came to an agreement. We also assessed risk of bias in the included studies in duplicate (PB and PW), using the PEDro scale for quality. This instrument has been shown to have acceptably high reliability and validity.^25^

### 2.7 Statistical analysis

Data analysis of efficacy was performed using statistical software provided by Revman5.3. Data analysis of safety was performed using statistical software provided by Stata16.0. For continuous variables, mean difference (MD) is adopted as the effective index, and the point estimated value and 95% confidence interval (CI) of each effect quantity are given. For the data of median, maximum, and minimum values mentioned in the included study, combined analysis is carried out after transformation according to the formula.^26^ The analysis was carried out for categorical variables using the risk ratio (RR), LogRR, and its 95% CI. The heterogeneity included in the study was analyzed by the X^2^ test (the test level was axiom 0.1) and evaluated with the I^2^ index. The random effect model is used for meta‐analysis regardless of the I^2^ index.

The sensitivity analysis was to remove the individual studies in turn, then to reconduct the meta‐analysis and evaluate the difference between the results after the exclusion and the original combined results. A *p*‐value of <0.05 was considered statistically significant.^24^

### 2.8 Nomenclature of targets and ligands

Key protein targets and ligands in this article are hyperlinked to corresponding entries in https://www.guidetopharmacology.org, the common portal for data from the IUPHAR/BPS Guide to PHARMACOLOGY,^27^ and are permanently archived in the Concise Guide to PHARMACOLOGY 2019/20.^28^

## 3 RESULTS

### 3.1 Study identification and selection

The database search identified 731 records by searching PubMed, Embase, Cochrane Central Register of Controlled Trials, Clinical trials, CNKI, Wanfang Data, VIP, CBM database dated until August 2021. After removing duplicates, 692 titles were initially screened, and nine theme‐related abstracts were selected for further screening. Four studies were excluded because data were not available. Finally, five studies were included in this systematic review (Figure 1).^1^, ^2^, ^11^, ^18^, ^29^ four used the values of the proportion of patients whose MRS score was 0–1 at day 90 in total, one used the mean difference in the change in MRS score at day 90, three used the change in NIHSS score at 24 h, two used the change in NIHSS score at day 7, two used the change in NIHSS score at day 90, two used the relative infarct lesion growth at 24 h, two used the relative infarct lesion growth at day 7.

### 3.2 Study characteristics and quality assessment

Table 1 lists detailed information from the five included studies. The included studies were published between 2014 and 2019. The number of participants per study ranged from 22 to 90, with a total number of 228. Patients who have received fingolimod were recorded in 114 of 228 (50%). All studies were randomized controlled trials.

**TABLE 1:** Clinical and demographic characteristics of 228 patients from five studies included in the systematic review

Reference (study)	Research type	Patient No	Country	Language	Interventions	Outcome measures
Zhang Liantao (2019)	RCTs	90	China	English	FTY720 ST	④⑦
De‐Cai Tian (2018)	RCTs	46	China	English	FTY720 ST	①②
Zilong Zhu (2015)	RCTs	47	China	English	FTY720 ST	①②⑤
Ying Fu (2014)	RCTs	22	China	English	FTY720 ST	①③④⑥
De‐Cai Tian (2017)	RCTs	23	China	Chinese	FTY720 ST	①②③⑤⑥

Note

RCTs: randomized clinical trials, FTY720:fingolimod, ST: standardized treatment, ① the proportion of patients whose MRS score was 0,1 at day 90, ② the change of NIHSS scores over 24 h, ③ the change of NIHSS scores at day 7, ④ the change of NIHSS scores at day 90, ⑤ relative infarct lesion growth over 24 h, ⑥ relative infarct lesion growth at day 7, ⑦ the mean difference in the change in MRS score at day 90.

Table 2 lists the characteristics of the patients in the included trials from the five included studies. The total number of patients is 228. There were 134 males and 94 females, with an average age of (63.125 ± 10.995) years.

**TABLE 2:** The characteristics of the patients in the included trials

		Age of participants		Male percentage	
Reference (study)	Patient no
Test group	Control group	Test group	Control group
Zhang Liantao (2019)^1^	90	63.31 ± 11.65	65.46 ± 11.70	31/45	26/45
De‐Cai Tian (2018)^11^	46	67 ± 6.8	65 ± 13	9/23	7/23
Zilong Zhu (2015)^18^	47	60 ± 12.5	59 ± 7.51	13/22	17/25
Ying Fu (2014)^44^	22	62.3 ± 8.0	54.7 ± 11.0	8/11	9/11
De‐Cai Tian (2017)^29^	23	66.2 ± 8.2	63.1 ± 11.1	7/13	7/10
Total	228	63.55 ± 10.5	62.7 ± 11.5	68/114	66/114

**FIGURE 2:**
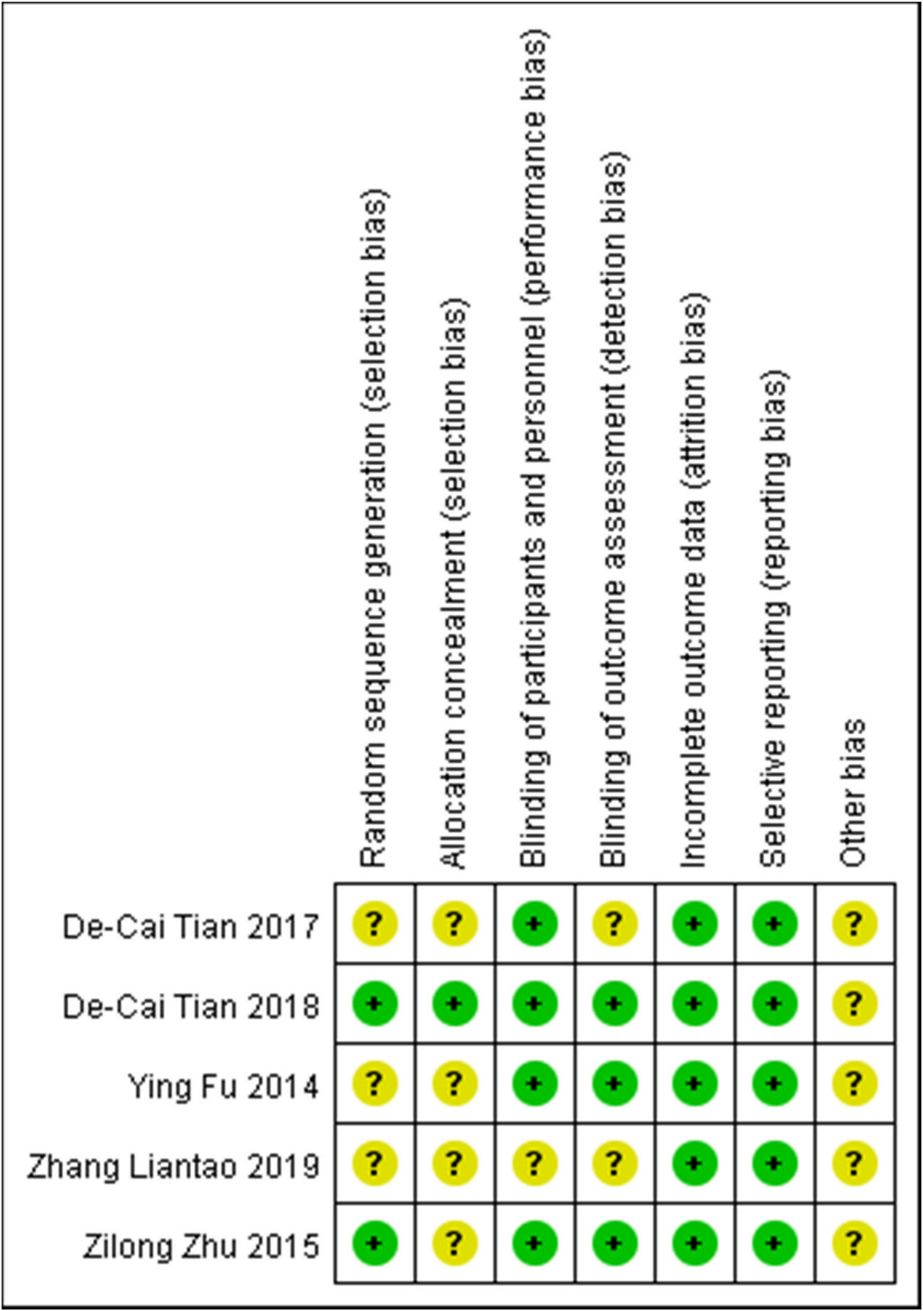
Risk of bias summary for included studies. A “+” stands for low risk, “–” for high risk, and “?” for unclear risk.

Figure 2 shows the risk of bias assessment of the five randomized trials; two trials described adequate methods of random sequence generation; one trial described allocation concealment. In four trials, the participants were blinded. The rate of dropout was low in all trials. None of these studies had incomplete outcome data or selective outcome reporting. All five studies had no other bias.

Table 3 lists the risk of bias in the included studies in duplicate (PB and PW), using the PEDro(The Physiotherapy Evidence Database)scale for quality.

**TABLE 3:** PEDro score of the trials included

	PEDro score										
Reference (study)	(1)	(2)	(3)	(4)	(5)	(6)	(7)	(8)	(9)	(10)	Total
ZhangLiantao (2019)	0	0	1	0	0	0	1	1	1	1	5
De‐Cai Tian (2018)	1	1	1	1	0	1	1	1	1	1	9
Zilong Zhu (2015)	1	0	1	1	0	1	1	1	1	1	8
Ying Fu (2014)	0	0	1	1	0	1	1	1	1	1	7
De‐Cai Tian (2017)	0	0	1	1	0	0	1	1	1	1	6

Note

PEDro: The Physiotherapy Evidence Database. The PEDro scale criteria are as follows: (1) random allocation, (2) concealed allocation, (3) baseline comparability, (4) blinding of patients, (5) blinding of therapist, (6) blinding of assessor, (7) adequate follow‐up, (8) intention‐to‐treat analysis, (9) between‐group comparison, (10) point estimate and variability. 0, absent; 1, present.

**FIGURE 3:**
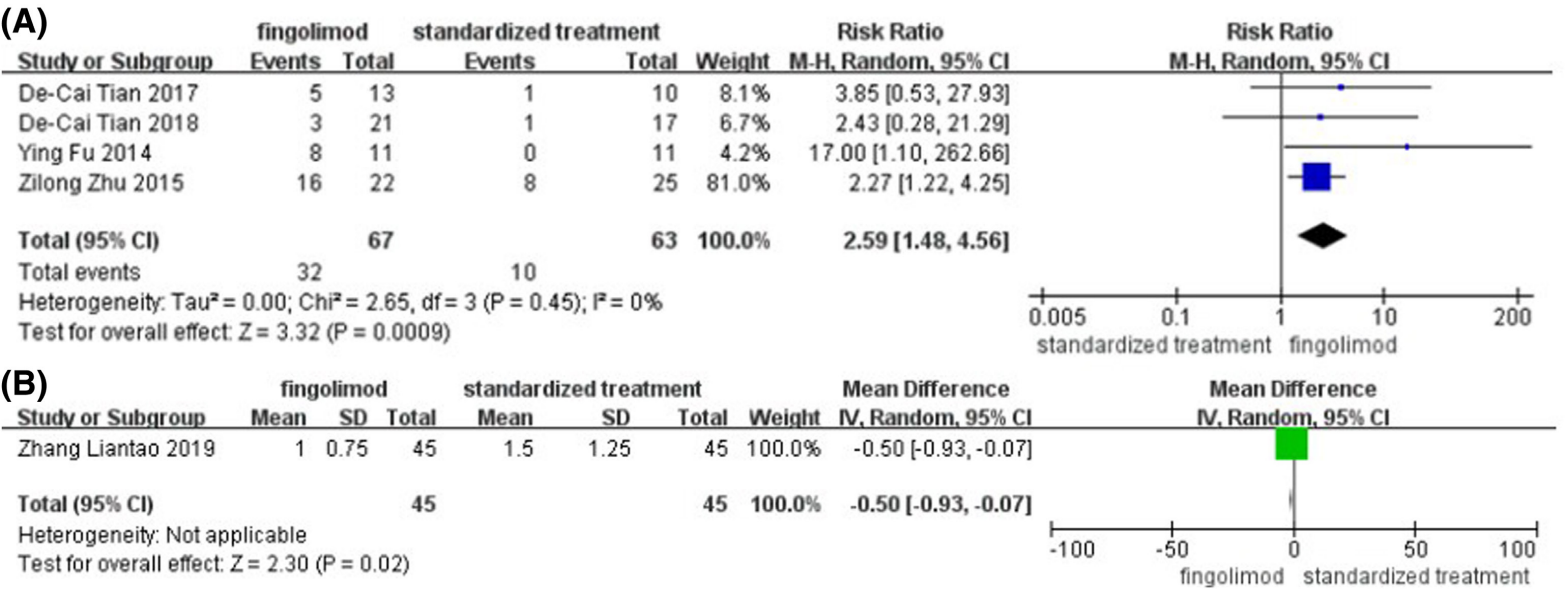
(A) Forest plot of the risk ratio of the proportion of patients whose MRS score was 0–1 at day 90, (B) the mean difference in the change in MRS score at day 90 between fingolimod plus standardized treatment and standardized treatment alone.

Figure 3, including five articles, shows a forest plot of the risk ratio of patients whose MRS score was 0–1 at day 90, the mean difference in the change in MRS score at day 90 between fingolimod plus standardized treatment, and standardized treatment alone. Figure 3A, including four articles, shows a forest plot of the risk ratio of the proportion of patients whose MRS score was 0–1 at day 90 between fingolimod plus standardized treatment and standardized treatment alone. This finding suggested that the risk ratio of the proportion of patients whose MRS score was 0–1 at day 90 between fingolimod plus standardized treatment and standardized treatment alone was 2.59 (95%CI, 1.48–4.56). A random‐effect model was used. Sensitivity analyses were performed by removing each study in turn and reanalyzed it. No studies were found to significantly affect heterogeneity. Figure 3B, including one article, shows a forest plot of the mean difference in the change in MRS score at day 90. The mean difference in MRS scores change at day 90 of fingolimod plus standardized treatment versus standardized treatment alone was −0.50 (95%CI, −0.93 to −0.07). A random‐effect model was used.

**FIGURE 4:**
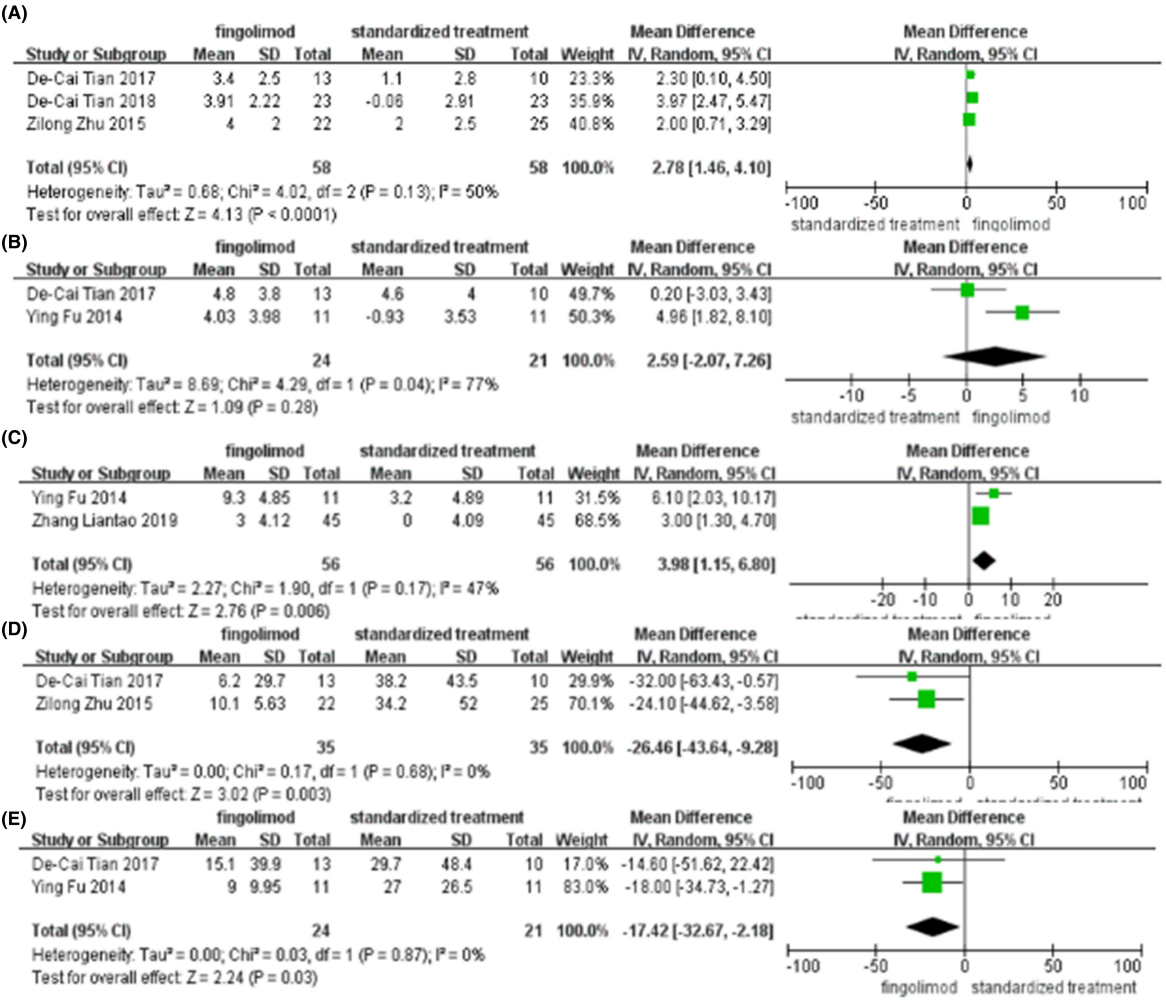
(A) Forest plot of the mean difference in the change in NIHSS score at 24 h, (B) NIHSS score at day 7, (C) CNIHSS score at day 90, (D) relative infarct lesion growth at 24 h, (E) relative infarct lesion growth at day 7 between fingolimod plus standardized treatment and standardized treatment alone.

Figure 4 shows a forest plot of the mean difference in the change in NIHSS score at 24 h, NIHSS score at day 7, NIHSS score at day 90, relative infarct lesion growth at 24 h, relative infarct lesion growth at day 7 between fingolimod plus standardized treatment and standardized treatment alone. Figure 4A, including three articles, shows a forest plot of the mean difference in the change in NIHSS score at 24 h between fingolimod plus standardized treatment and standardized treatment alone. This finding suggested that the mean difference in NIHSS score change at 24 h of fingolimod plus standardized treatment versus standardized treatment alone was 2.78 (95%CI, 1.46–4.10). A random‐effect model was used. Sensitivity analyses were performed by removing each study in turn and reanalyzed it. The application of sensitivity analysis showed that the study by De‐Cai Tian et al. (2017) significantly affected heterogeneity. Figure 4B, including two articles, shows a forest plot of the mean difference in the change in NIHSS score at day 7 between fingolimod plus standardized treatment and standardized treatment alone. This finding suggested that the mean difference in NIHSS score change at day 7 of fingolimod plus standardized treatment versus standardized treatment alone was 2.59 (95%CI, −0.27 to 7.26). A random‐effect model was used. Figure 4C, including two articles, shows a forest plot of the mean difference in the change in NIHSS score at day 90 between fingolimod plus standardized treatment and standardized treatment alone. This finding suggested that the mean difference in NIHSS score change at day 90 of fingolimod plus standardized treatment versus standardized treatment alone was 3.98(95%CI, 1.15–6.80). A random‐effect model was used. Figure 4D, including two articles, shows a forest plot of the mean difference in the change in relative infarct lesion growth at 24 h between fingolimod plus standardized treatment and standardized treatment alone. This finding suggested that the mean difference in relative infarct lesion growth change at 24 h of fingolimod plus standardized treatment versus standardized treatment alone was −26.46 (95%CI, −43.64 to −9.28). A random‐effect model was used. Figure 4E, including two articles, shows a forest plot of the mean difference in the change in relative infarct lesion growth at day 7 between fingolimod plus standardized treatment and standardized treatment alone. This finding suggested that the mean difference in relative infarct lesion growth change at day 7 of fingolimod plus standardized treatment versus standardized treatment alone was −17.42 (95%CI, −32.67–−2.18). A random‐effect model was used.

### 3.3 Safety outcomes

We combined the data retrieved from the five trials for serious adverse events (SAEs) and adverse events (AEs) such as deaths, myocardial infarctions, recurrent strokes, hernia, hemorrhage of the digestive tract, fever (>38°C), hemorrhagic transformation at 24 h, lung infection, urinary tract infection, herpes virus infection, abnormal laboratory liver function test, gastrointestinal disorders, arrhythmia, and macular edema. The collected data of common AEs are displayed in Table 4. Data analysis was performed using statistical software provided by Stata16.0. We did not find any significant difference between the fingolimod and standardized treatment groups in terms of SAEs and AEs.

**TABLE 4:** Safety outcomes in the meta‐analysis

	No. of studies	LogRR	95%CI	*p* value
Complications				
Deaths	5	–1.08	–2.59–0.43	0.16
Myocardial infarctions	5	0.28	–1.37–1.92	0.74
Recurrent strokes	5	0.26	–1.39–1.91	0.75
Hernia	5	–0.97	–2.02–0.07	0.07
Hemorrhage of the digestive tract	5	–0.72	–2.00–0.56	0.27
Hemorrhagic transformation at 24 h	2	0.94	–0.20–2.08	0.11
Fever (>38°C)	4	–0.09	–0.89–0.71	0.82
Event				
All events				
At least one adverse event	3	–0.12	–0.85–0.61	0.75
Any serious adverse event	4	–0.06	–1.99–1.87	0.95
Frequent or special interest adverse events				
Lung infection	5	0.06	–0.58–0.69	0.86
Urinary tract infection	5	0.02	–0.95–0.99	0.97
Abnormal laboratory liver function test	3	–0.04	–2.26–2.18	0.97
Gastrointestinal disorders	3	–0.04	–2.26–2.18	0.97
Herpes virus infection	4	–0.03	–1.96–1.90	0.98
Arrhythmia	3	0.67	–1.33–2.66	0.51
Macular edema	3	–0.04	–2.26–2.18	0.97

Abbreviations: RR, relative risk; CI, confidence interval.

## 4 DISCUSSION

This meta‐analysis included five trials to assess the efficacy and safety of fingolimod in patients with AIS. Recently, the effectiveness and safety of fingolimod in patients with AIS have been investigated in some RCTs.^1^, ^2^, ^11^, ^18^, ^29^ This systematic review and meta‐analysis provide data to support the efficacy and safety of fingolimod for AIS.

### 4.1 The efficacy of Fingolimod

Our meta‐analysis presented that fingolimod resulted in the decrease of infarct growth and improved clinical function. Our primary endpoint here is based on a proportion of patients with MRS 0–1 at 90 days, decrease in NIHSS score at 24 h, decrease in NIHSS score at day 7, decrease in NIHSS score at 90 days, relative infarct lesion growth at 24 h, and relative infarct lesion growth at 7 days. The sensitivity analysis with analyses of the decrease in NIHSS score at 24 h and at day 7 showed that the study by De‐Cai Tian et al. (2017) significantly affected heterogeneity. It showed no statistically significant differences between fingolimod and standardized treatment in NIHSS score at day 7.

Studies have shown critical linkages between various immunomodulatory mechanisms in ischemic stroke.^18^, ^30^ Ischemic stroke involves neuronal dysfunction and complex interactions between other cells, including vascular endothelial cells, BBB, extracellular matrix, and immune system.^31^, ^32^, ^33^ Early clinical observations suggest a link between inflammation and ischemic stroke. Inflammation predisposes people to ischemic stroke and directly leads to many pathological changes.^32^, ^34^, ^35^, ^36^, ^37^ Further understanding of the relationship between immunity and brain tissue in ischemic stroke is helpful to develop new immunomodulatory therapy.

Fingolimod significantly reduced infarct expansion at 24 h. Fingolimod not only inhibits lymphocyte infiltration into the brain parenchyma and protects brain tissue from secondary injury but also, at an earlier stage, by reducing the number of cells accumulating in the brain microvasculature. Inhibit the formation of capillary‐inflammatory thrombosis and protect the function of the CNS.^38^, ^39^, ^40^, ^41^ In addition, fingolimod also targets intrinsic cells of the CNS, including vascular endothelial cells. It produces nonimmune effects, thereby protecting brain tissue to some extent. The effect of fingolimod on vascular endothelial cells can inhibit the proinflammatory and thrombotic states of endothelial cells and improve the integrity of BBB.^38^, ^42^

### 4.2 The safety of Fingolimod

Our meta‐analysis showed no significant difference in the incidence of complications and adverse events between fingolimod and the standard treatment. Because of the brief fingolimod treatment, this drug does not necessarily produce an immune‐deficient state.

### 4.3 Strengths and limitations

Our meta‐analysis aimed to present efficacy and safety data on humans. To date, there was a previous meta‐analysis of 17 experimental articles on fingolimod (580 animals), and the main goal was to update the evidence how fingolimod affects experimental stroke.^43^ No meta‐analysis has been published about the effects and safety of fingolimod for AIS on humans. Limitations of this study: (1) Although the search strategy is relatively complete, it does not rule out that eligible articles are not included. (2) A large sample of studies lacked in the included studies. (3) The fact that it only includes randomized controlled trials. (4) It is not distinguished patients who receive different standard treatments such as the intravenous administration of tPA, intravascular therapy, antiplatelet drugs, and so on. (5) Four of the included trials came from the same group of investigators. (6) Four of the excluded studies’ data are not extractable. The records with unobtainable data may cause bias in the results. (7) None of the included trials were double‐blinded (most had a PROBE design). (8) High heterogeneity across studies should not be neglected, though a random‐effects model was used for adjustment. Nonetheless, results were broadly similar even if sensitivity analysis which decreased the heterogeneity were performed. Inherent limitations in the majority of meta‐analyses, such as lack of access to raw data and the variety in definitions of outcomes in the included studies, are unavoidable. None of the included studies was adequately sized to evaluate the proposed primary endpoint. (9) The entire data were derived from patients in China. More studies are needed that include other ethnic groups. (10) Different inclusion/exclusion criteria and follow‐up periods in the included studies led to high heterogeneity. (11) The treatment of five included studies did not cover intravascular therapy. (12) Five studies included only phase 1 or early phase 2 trials with very small sample sizes. (13) The quality of studies is poor. (14) The sample sizes for each of the analysis are small. (15) Four five studies are from the same group and all from China. RCTs with greater patient numbers will be needed for future studies. (16) The method for infarct growth measured in the trials was not uniformity.

## 5 CONCLUSION

The Fingolimod plus standard treatment group decreased infarct growth and improved clinical function than the standard treatment. There was no significant difference in the incidence of complications and adverse events between the standard treatment group and fingolimod plus standard treatment group. Our study shows that these early results are promising; larger studies in different patient populations are needed to validate the studies.

## DISCLOSURE

The authors declare no conflict of interest.

## 6 ETHICS APPROVAL STATEMENT

As this was a systematic review, no ethics approval was sought.

## AUTHOR CONTRIBUTIONS

PB, PW, FJ, BL, ML designed the study, searched and screened the literature, extracted and analyzed the data, drafted the manuscript. PB, JZ, YY, NL collated the data, guided manuscript writing. JY, CZ, ZL, MW revised the manuscript.

## 7 DATA AVAIL ABILITY STATEMENT

Data sharing is not applicable to this article as no new data were created or analyzed in this study.

## Supporting information

Appendix S1

Appendix S2
