# Supplementary material for: The efficacy and safety of fingolimod plus standardized treatment versus standardized treatment alone for acute ischemic stroke: A systematic review and meta‐analysis
Source: Pharmacol Res Perspect. 2022 May 18;10(3):e00972. doi: 10.1002/prp2.972 (PMC9117458; doi:10.1002/prp2.972)
Supplement: Supplementary file 1 — Appendix S1 [file PRP2-10-e00972-s001.docx]

Pubmed：

#1 MeSH descriptor: [Cerebrovascular Disorders] explode all trees

#2 (stroke* or cva* or cerebrovasc* or "cerebral vascular*" or poststroke or post‐stroke):ti,ab,kw (Word variations have been searched)

#3 (cerebral or cerebellar or brain* or vertebrobasilar):ti,ab,kw (Word variations have been searched)

#4 (infarct* or ischemi* or ischaemi* or thrombo* or emboli* or apople*):ti,ab,kw (Word variations have been searched)

#5 (#3 and #4)

#6 (cerebral or intracerebral or intracranial or brain* or subarachnoid):ti,ab,kw (Word variations have been searched)

#7 (haemorrhage or hemorrhage or bleed*):ti,ab,kw (Word variations have been searched)

#8 (#6 and #7)

#9 MeSH descriptor: [Hemiplegia] this term only

#10 MeSH descriptor: [Brain Injuries] this term only

#11 MeSH descriptor: [Aphasia] explode all trees

#12 MeSH descriptor: [Dysarthria] this term only

#13 MeSH descriptor: [Apraxias] this term only

#14 MeSH descriptor: [Deglutition Disorders] this term only

#15 (hemipleg* or hemipar*):ti,ab,kw (Word variations have been searched)

#16 (aphasi* or dysphasi* or dysarthri* or dysphag* or aprax* or dysprax*):ti,ab,kw (Word variations have been searched)

#17 (swallow*):ti,ab,kw (Word variations have been searched)

#18 (impair* or disorder* or problem* or difficult*):ti,ab,kw (Word variations have been searched)

#19 #17 and #18

#20 ("unilateral neglect" or "neglect syndrome*" or "visual neglect" or hemianop*):ti,ab,kw (Word variations have been searched)

#21 (#1 or #2 or #5 or #8 or #9 or #10 or #11 or #12 or #13 or #14 or #15 or #16 or #19 or #20)

#22 MeSH Fingolimod Hydrochloride

#23 fingolimod OR FTY720 OR "FTY 720" OR "fingolimod hydrochloride" OR FTY‐720 OR “2‐amino‐2‐(2‐(4‐octylphenyl)ethyl)‐1,3‐propanediol hydrochloride" OR Gilenya OR “sphingosine fosphate receptor antagonist” OR Gilenia

#24 #22 OR #23

#25 #21 AND #24

#26 ("Randomized Controlled Trial" [Publication Type] OR "Controlled Clinical Trial" [Publication Type] OR "Clinical Trials as Topic"[Mesh:NoExp] OR randomized[Title/Abstract] OR placebo [Title/Abstract] OR randomly[Title/Abstract] OR trial[Title/Abstract]) NOT ("Animals"[Mesh] NOT "Humans"[Mesh])

#27 #25 AND #26

Embase:

1. exp cerebrovascular disease/

2. (stroke$ or cva$ or cerebrovasc$ or "cerebral vascular$" or poststroke or post‐stroke).tw.

3. (cerebral or cerebellar or brain$ or vertebrobasilar).tw.

4. (infarct$ or isch?emi$ or thrombo$ or emboli$ or apople$).tw.

5. 3 and 4

6. (cerebral or intracerebral or intracranial or brain$ or subarachnoid).tw.

7. (haemorrhage or hemorrhage or bleed$).tw.

8. 6 and 7

9. brain injury/

10. hemiplegia/

11. aphasia/

12. dysarthria/

13. apraxia/

14 "apraxia of speech"/

15. dysphagia/

16. (hemipleg$ or hemipar$).tw.

17. (aphasi$ or dysphasi$ or dysarthri$ or dysphag$ or aprax$ or dysprax$).tw.

18. (swallow$).tw.

19.(impair$ or disorder$ or problem$ or difficult$).tw.

20.18 and 19

21. ("unilateral neglect" or "neglect syndrome$" or "visual neglect$" or hemianop$).tw.

22. 1 or 2 or 5 or 8 or 9 or 10 or 11 or 12 or 13 or 14 or 15 or 16 or 17 or 20 or 21

23. fingolimod/ exp

24. fingolimod OR FTY720 OR "FTY 720" OR "fingolimod hydrochloride" OR FTY‐720 OR “2‐amino‐2‐(2‐(4‐octylphenyl)ethyl)‐1,3‐propanediol hydrochloride" OR Gilenya OR “sphingosine fosphate receptor antagonist” OR Gilenia

25.23 or 24

26.25 and 22

27. 'crossover procedure':de OR 'double-blind procedure':de OR 'randomized controlled trial':de OR 'single-blind procedure':de OR (random* OR factorial* OR crossover* OR cross NEXT/1 over* OR placebo* OR doubl* NEAR/1 blind* OR singl* NEAR/1 blind* OR assign* OR allocat* OR volunteer*):de,ab,ti

28.26 and 27

Cochrane library(581)：Cochrane Central Register of Controlled Trials (6):

#1 MeSH descriptor: [Cerebrovascular Disorders] explode all trees

#2 (stroke* or cva* or cerebrovasc* or "cerebral vascular*" or poststroke or post‐stroke):ti,ab,kw (Word variations have been searched)

#3 (cerebral or cerebellar or brain* or vertebrobasilar):ti,ab,kw (Word variations have been searched)

#4 (infarct* or ischemi* or ischaemi* or thrombo* or emboli* or apople*):ti,ab,kw (Word variations have been searched)

#5 (#3 and #4)

#6 (cerebral or intracerebral or intracranial or brain* or subarachnoid):ti,ab,kw (Word variations have been searched)

#7 (haemorrhage or hemorrhage or bleed*):ti,ab,kw (Word variations have been searched)

#8 (#6 and #7)

#9 MeSH descriptor: [Hemiplegia] this term only

#10 MeSH descriptor: [Brain Injuries] this term only

#11 MeSH descriptor: [Aphasia] explode all trees

#12 MeSH descriptor: [Dysarthria] this term only

#13 MeSH descriptor: [Apraxias] this term only

#14 MeSH descriptor: [Deglutition Disorders] this term only

#15 (hemipleg* or hemipar*):ti,ab,kw (Word variations have been searched)

#16 (aphasi* or dysphasi* or dysarthri* or dysphag* or aprax* or dysprax*):ti,ab,kw (Word variations have been searched)

#17 (swallow*):ti,ab,kw (Word variations have been searched)

#18 (impair* or disorder* or problem* or difficult*):ti,ab,kw (Word variations have been searched)

#19 #17 and #18

#20 ("unilateral neglect" or "neglect syndrome*" or "visual neglect" or hemianop*):ti,ab,kw (Word variations have been searched)

#21 (#1 or #2 or #5 or #8 or #9 or #10 or #11 or #12 or #13 or #14 or #15 or #16 or #19 or #20)

#22 MeSH Fingolimod Hydrochloride

#23 fingolimod OR FTY720 OR "FTY 720" OR "fingolimod hydrochloride" OR FTY‐720 OR 2‐amino‐2‐(2‐(4‐octylphenyl)ethyl)‐1,3‐propanediol hydrochloride OR Gilenya OR “sphingosine fosphate receptor antagonist” OR Gilenia

#24 #22 OR #23

#25 #21 AND #24

Clinical trials:

Condition or disease: Cerebrovascular Disorders

Other terms: fingolimod

CNKI:

(SU % '卒中' OR SU % '脑损伤') AND (SU % '芬戈莫德'OR SU % '免疫调节剂')

Wanfang Data:

(主题:（卒中） + 主题:(脑损伤)) * (主题:(芬戈莫德） + 主题:(免疫调节剂）)

VIP:

(M=(卒中 OR 脑损伤) OR R=(卒中 OR 脑损伤)) AND (M=(芬戈莫德 OR 免疫调节剂) OR R=(芬戈莫德 OR 免疫调节剂))

CBM:

("芬戈莫德"[摘要:智能] OR "免疫调节剂"[摘要:智能]) AND ("卒中"[摘要:智能] OR "脑损伤"[摘要:智能])
