# Supplementary material for: The efficacy and safety of fingolimod plus standardized treatment versus standardized treatment alone for acute ischemic stroke: A systematic review and meta‐analysis
Source: Pharmacol Res Perspect. 2022 May 18;10(3):e00972. doi: 10.1002/prp2.972 (PMC9117458; doi:10.1002/prp2.972)

| **Section/topic** | **#** | **Checklist item** | **Reported on page #** |
| --- | --- | --- | --- |
| **TITLE** | | |  |
| Title | 1 | The report is identified as a meta-analysis | 1 |
| **ABSTRACT** | | |  |
| Structured summary | 2 | The structured abstract includes Background and Purpose, Methods, Results and Conclusion. | 2 |
| **INTRODUCTION** | | |  |
| Rationale | 3 | Described in the introduction. | 2 |
| Objectives | 4 | Stated in the introduction. | 3 |
| **METHODS** | | |  |
| Protocol and registration | 5 | The protocol is described in the Methods. Our protocol was registered prospectively with the Prospero website(CRD42021272343).the international prospective register of systematic reviews available at https:// www.crd.york.ac.uk/prospero/display_record.php?ID=CRD42021272343. | 3 |
| Eligibility criteria | 6 | Described in the Methods. | 3 |
| Information sources | 7 | Described in the Methods. | 3 |
| Search | 8 | Described in the Methods. | 3 |
| Study selection | 9 | Described in the Methods. | 3-4 |
| Data collection process | 10 | Described in the Methods. | 4 |
| Data items | 11 | Described in the Methods. | 4 |
| Risk of bias in individual studies | 12 | Described in the Methods. | 4 |
| Summary measures | 13 | Described in the Methods. | 4-5 |
| Synthesis of results | 14 | Described in the Methods. | 5 |

Page 1 of 2

| **Section/topic** | **#** | **Checklist item** | **Reported on page #** |
| --- | --- | --- | --- |
| Risk of bias across studies | 15 | Because less than 10 studies were included, risk of bias across studies was not carried out. |  |
| Additional analyses | 16 | Described in the Methods. | 5 |
| **RESULTS** | | |  |
| Study selection | 17 | Described in the results. | 5 |
| Study characteristics | 18 | Described in the results. | 5-6 |
| Risk of bias within studies | 19 | Described in the results. | 5 |
| Results of individual studies | 20 | Described in the results. | 5-7 |
| Synthesis of results | 21 | Described in the results. | 5-7 |
| Risk of bias across studies | 22 | Because less than 10 studies were included, risk of bias across studies was not carried out. |  |
| Additional analysis | 23 | Described in the results | 5-7 |
| **DISCUSSION** | | |  |
| Summary of evidence | 24 | Described in the discussion. | 7-8 |
| Limitations | 25 | Described in the discussion. | 8 |
| Conclusions | 26 | Described in the discussion | 8 |
| **FUNDING** | | |  |
| Funding | 27 | This work was also funded by the Doctoral Research start-up fund of Inner Mongolia Autonomous Region People’s Hospital(Grant No. 2021BS04),the Natural Science Foundation of Inner Mongolia Autonomous Region of China (Grant No. 2020MS03063), the Natural Science Foundation of Inner Mongolia Autonomous Region of China(Grant No.2018MS8009), and the Natural Science Foundation of Inner Mongolia Autonomous Region of China(Grant No.2019MS08077). | 1 |

*From:*  Moher D, Liberati A, Tetzlaff J, Altman DG, The PRISMA Group (2009). Preferred Reporting Items for Systematic Reviews and Meta-Analyses: The PRISMA Statement. PLoS Med 6(6): e1000097. doi:10.1371/journal.pmed1000097

For more information, visit: **www.prisma-statement.org**.

Page 2 of 2


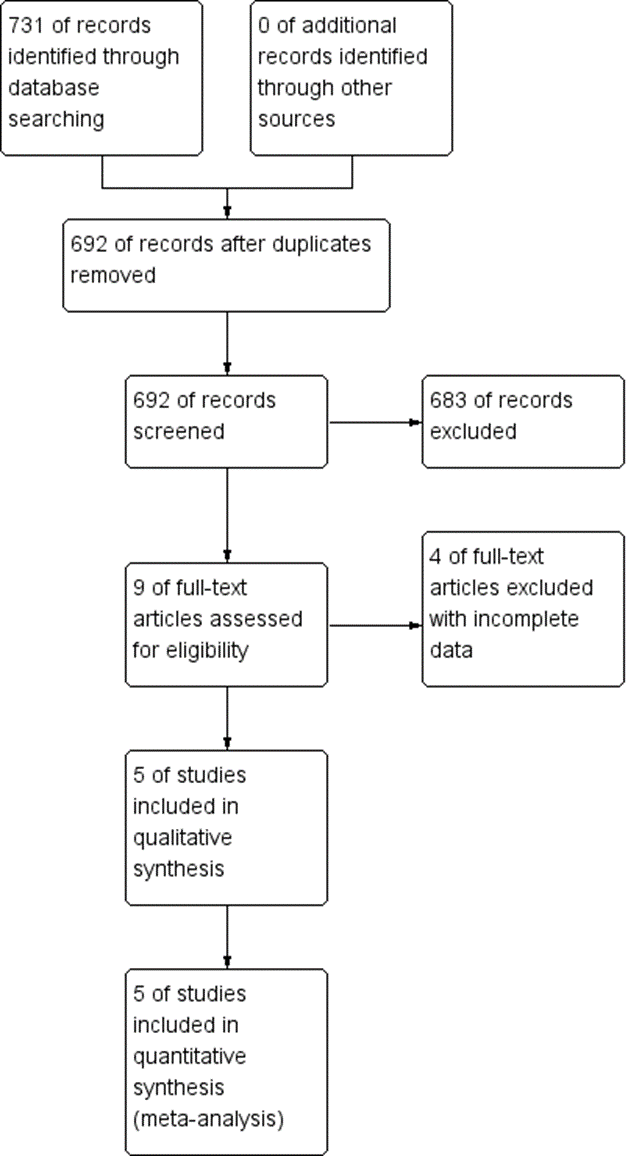

Supplement: Supplementary file 2 — Appendix S2 [file PRP2-10-e00972-s002.docx]
